# Selective impairment and a positive recognition bias of the facial emotion recognition after propofol anesthesia during gastrointestinal endoscopy

**DOI:** 10.3389/fpsyg.2025.1691042

**Published:** 2026-01-13

**Authors:** Zhuonan Sun, Qiong Lan, Hua Zhang, Lijing Zheng, Qingao Liu, Haoyu Zuo, Yu Feng, Yusen Xiao, Ning Yang, Xixi Jia, Yanan Song, Yajie Liu, Dengyang Han, Yinyin Qu, Jing Zhang, Ye Wang, Zhengqian Li, Xiangyang Guo, Taotao Liu

**Affiliations:** 1 Department of Anesthesiology, Peking University Third Hospital, Beijing, China; 2 Research Center of Clinical Epidemiology, Peking University Third Hospital, Beijing, China; 3 Department of Biomedical Informatics, School of Basic Medical Sciences, Peking University Health Science Center, Beijing, China; 4 Department of Obstetrics and Gynecology, Peking University Third Hospital, Beijing, China; 5 Department of Orthopedic, Peking University Third Hospital, Beijing, China; 6 Department of Gastroenterology, Peking University Third Hospital, Beijing, China

**Keywords:** anesthesia, propofol, facial emotion recognition, delay discounting, gastrointestinal endoscopy

## Abstract

**Introduction:**

Propofol may induce emotional impairment like euphoria and elation. Previous studies have demonstrated that emotional impairment can injure social cognition like emotion recognition and decision-making abilities. Therefore, this study is designed to investigate the effects of propofol anesthesia on facial emotion recognition (FER) and delay discounting behavior.

**Method:**

Patients underwent diagnostic gastrointestinal endoscopy (GI) with propofol anesthesia in this prospective cohort observational study. Prior to and following the procedure (approximately 30 min afterwards), patients were asked to select the word that best describes the presented facial photographs displaying happiness, anger, and neutral expressions. Additionally, Monetary Choice Questionnaire-9 was used to assess delay discounting.

**Results:**

Within a cohort of 87 patients, 11 patients (12.6%) met the criterion of FER deficit post-GI. The FER of anger exhibited significant differences between pre- and post-GI, considering both the correct (52.6%) and incorrect (24.3%) recognition. There was a positive identification bias for FER after propofol anesthesia: mistaking anger (*p* = 0.02) or neutral (*p* = 0.01) expression for happiness. Procedures in the morning and the absence of insomnia were associated with the decreased FER score of anger post-GI. The results did not indicate any impairment of propofol anesthesia on FER of happiness or delay discounting behavior.

**Conclusion:**

The study demonstrates that propofol anesthesia during GI endoscopy selectively impairs the recognition of anger facial expressions while leaving the recognition of happiness and delay discounting unaffected at a short-term postoperative observation. Additionally, the recognition of anger and neutral facial expressions exhibited a tendency towards a positive bias.

**Clinical trial registration:**

https://www.chictr.org.cn/showproj.html?proj=199458, identifier ChiCTR2300073132.

## 1 Introduction

Propofol, a widely used intravenous sedative or anesthetic, is characterized by its quick onset, fleeting half-life, and safety profiles in procedural sedation, monitored anesthesia care, and general anesthesia. Due to its fast recovery with fewer side effect, propofol is recommended in guidelines for sedation in ambulatory surgery in outpatients, such as painless procedures (Lee and Lee, 2014).

An increasing body of evidence indicates propofol’s effect on affective and reward processes. Patients who underwent painless procedures (gynecological endoscopy, gastrointestinal endoscopy, or colonoscopy) experienced pleasant effects upon awakening from propofol anesthesia, including elation, euphoria (Monedero Rodríguez et al., 1991; Zhao et al., 2023), pleasant dreams (Monedero Rodríguez et al., 1991; Tezcan et al., 2014), and a general sensation of well-being (Kim et al., 2015). Furthermore, healthy volunteers felt elevated mood or became talkative when subtherapeutic doses of propofol were used. Animal studies have demonstrated that propofol induces a pleasant affective state in the place-conditioning paradigm, both at subtherapeutic doses and after recovery from propofol anesthesia (Pain et al., 1996, 1997). A study also showed that propofol, both at subtherapeutic and anesthesia doses, significantly elevated dopamine levels within the nucleus accumbens (Pain et al., 2002), a crucial brain region responsible for emotional regulation and drug addiction. These findings suggest that propofol may exert an influence on emotional processes.

Emotional impairment may impair social cognition. Social cognition refers to one’s capacity to comprehend and process the emotions, intentions and behaviors of others within social contexts. It constitutes one of the six fundamental abilities of neural cognition. Serving as the foundation and prerequisite for social interaction, social cognition is closely related to social functioning and mental health. Social cognitive impairment can detrimentally affect interpersonal communication skills, eliminate perception of social support, and consequently reduce social interaction, potentially leading to a vicious cycle of social communication disorders. Social cognitive impairment is recognized as early and significant characteristics of various mental disorders (such as depression) and neurodegenerative diseases (such as AD and Parkinson’s disease). Therefore, research on social cognition is of great significance in fields such as psychology, neuroscience and psychiatry.

Social cognitive processes can be clustered in three domains associated with social perception, social understanding and social decision-making. Facial emotion recognition (FER) plays a significant role in interpersonal engagement and social perception. Many neuropsychological diseases exhibit impairments in FER and demonstrate biased facial emotion detection. Research indicated that individuals with major depression disorder tend to exhibit a diminished capacity in processing facial emotion expressions (Gao et al., 2019; Ruhe et al., 2019). Similarly, patients with anxiety, bipolar disorder or schizophrenia also display deficits in FER (Tseng et al., 2017; Isik Ulusoy et al., 2020; Kuang et al., 2022). Impaired FER is also a common occurrence that poses obstacles to the social cognition of individuals with drug addiction and dementia (Castellano et al., 2015; McLellan et al., 2008; Pietschnig et al., 2016).

Delay discounting behavior, characterized by the preference for an immediate smaller reward over a delayed larger reward, is a prevalent phenomenon among human beings and holds significant importance in the realms of social decision-making (Mitchell and Wilson, 2012; Mischel et al., 1989). The choices made in delay discounting can be influenced by biological or pathological factors. Notably, intertemporal choice exhibits reduced discounting for longer time periods when individuals are in a positive mood (Beukeboom and Semin, 2005; Forgas and East, 2008). Conversely, individuals experiencing sleep deprivation tend to show steeper discounting rates (Reynolds and Schiffbauer, 2004). Moreover, individuals with major depression disorder display higher rates of discounting (Pulcu et al., 2014). Higher rates of discounting may also exhibit a consistent correlation with indicators of substance misuse or tobacco consumption (Mitchell and Wilson, 2012; Pritschmann et al., 2021).

While many studies have explored the general cognitive dysfunction after propofol anesthesia, very few studies have drawn attention to social cognition in the postoperative period. Perioperative social cognitive abilities are of profound influence on the perioperative management. For example, impairment on emotional recognition may lead to misunderstanding of medical staff. Deficits in decision-making may affect the evaluation of the risks with surgery and medical treatment (Frietsch et al., 2014). Such misjudgement can increase medical complications and disputes, thereby escalating healthcare resource utilization and financial burdens. Recently, two studies focused on the emotion recognition impairment after cardiac surgeries with cardiopulmonary bypass utilizing propofol anesthesia (Zhang et al., 2020, 2022). The results revealed that 18.35%–31.25% of patients displayed deficits in emotion recognition 7 days after surgery, although further investigation on the potential reason or the expression type of emotion recognition deficit is lacking. There is currently a lack of research investigating delay discounting after anesthesia/surgery.

It has been observed that propofol-induced euphoria is more frequently reported among patients undergoing brief or minimally invasive procedures, such as gynecological, oocyte pick-up, or gastrointestinal endoscopy (Monedero Rodríguez et al., 1991; Tezcan et al., 2014). Moreover, daytime examinations and procedures are performed in outpatient settings, after which patients are typically discharged shortly after recovery. This may leave an impression to the patients that their cognitive functions have completely recovered, enabling them to safely resume routine daily activities and even engage in critical decision-making tasks, such as contract negotiations or important meetings. This widespread but unverified assumption underscores the necessity to objectively evaluate whether social cognitive functions, crucial for interpersonal interactions and complex judgments, are indeed preserved after propofol anesthesia. Therefore, it was hypothesized that propofol anesthesia may have a detrimental impact on FER and delay discounting in patients undergoing brief or minimally invasive procedures. Accordingly, this study was designed to assess the emotion recognition abilities and delay discounting behavior in patients undergoing gastrointestinal endoscopy (GI) with propofol anesthesia. The objective of this study was to ascertain whether propofol has any discernible effects on social cognitive functions.

## 2 Materials and methods

A prospective single-center, cohort study was conducted at the Endoscopic Ambulatory Surgery Centers in Peking University Third Hospital from July to September 2023. The primary objective was to assess the potential impairment of FER following propofol anesthesia during GI. Additionally, secondary objectives included investigating the effects of propofol anesthesia on delay discounting, dream conditions, sense of well-being, as well as identifying factors that may contribute to FER impairment.

### 2.1 Participants

Adult patients (aged 18–65 years) who underwent diagnostic esophagogastroduodenoscopy and colonoscopy with American Society of Anesthesiologists (ASA) grade 1–2 (ASA I, indicating an otherwise healthy patient except for the surgery; ASA II, indicating a patient with mild systemic disease with good tolerance) were included. Patients who met any of the following criteria were excluded from the study: documented allergic reaction to propofol or opioids, misuse of opioids or propofol, history of cognitive impairment (such as dementia or Parkinson’s disease), inability to communicate due to language barrier, speech disorder, severe hearing or vision impairment, the requirement for therapeutic procedures during GI or anesthesia and surgery lasting longer than 60 min, failing for colonoscopy due to inadequate bowel preparation, and unwillingness to participate in the research. Due to the geographical location of the hospital, all patients included in this study were Asian.

### 2.2 Materials

#### 2.2.1 Facial emotion recognition (FER)

Several tests are employed to assess FER, with adjustments commonly made to cater to specific research objectives. Comparing to the world-wide used Ekman test, consisting of 60 photographs with 6 emotions: happiness, anger, disgust, surprise, sadness, and fear, this study focused on two emotions-happiness (a positive emotion) and anger (a negative emotion)—rather than other emotions. This decision was based on previous research indicating that happiness exhibits greater stability in FER within neuropsychological contexts while anger is more susceptible to impairment (Guaita et al., 2009; Sato et al., 2002; Ciccarelli et al., 2022; da Silva Ferreira et al., 2014). Additionally, despite the lack of existing FER studies examining the effects of propofol anesthesia, it has been reported that propofol induces feelings of “elation” or “euphoria” upon awakening (Monedero Rodríguez et al., 1991; Zhao et al., 2023). Each emotion was represented by three distinct photographs, each sourced from a different individual. This limited number of stimuli was deemed advantageous and practicable within the fast-paced environment of Endoscopic Ambulatory Surgery Centers in our institution.

This study employed a tablet-based Facial Expression Recognition task. Prior to the administration of the task, patients were shown two examples without time restraint, from a male and a female, each displaying two emotional faces (happiness and anger) and a neutral expression. There was no time constraint imposed during this familiarization phase. Subsequently, nine grayscale photographs featuring neutral expressions and two emotional expressions (happiness and anger) were presented randomly on the tablet screen. Each face was displayed for 1 s, followed by a background screen displaying visual white noise. During this interval, participants were instructed to select the most appropriate emotion label from three options (Happiness, Anger, or Neutral) using a provided form. There was no time limit for responding and no feedback given during the experiment. A correct answer was assigned a score of 1, while an incorrect answer received a score of 0. The scoring system ranged from 0 to 3 for each expression.

The one standard deviation (1 SD) rule was adopted for assessing FER. This method has been extensively employed in clinical research to assess cognitive or psychological decline during repeated testing, due to its advantage of reducing false positives (Zhang et al., 2020, 2022). According to the 1 SD rule, a decline in performance that surpasses 1 SD from the group’s baseline performance is considered a “true” deterioration. Therefore, in this study, the FER difference between the pre-operative score and post-operative score was calculated (score _pre−operative_—score _post−operative_). If a patient’s score decreased by more than 1 SD of pre-operative FER scores of all patients, that patient was classified as having FER impairment.

To account for the facial characteristic differences between Caucasians and Mongolians, the photographs utilized in this test were sourced from the CAS-PEAL Database (Wen Gao et al., 2008), which offers a comprehensive collection of Mongolian faces. To determine the faces to be used for testing, a preliminary screening process was conducted involving healthy volunteers. A group of 30 healthy college students (18 males and 12 females, with a mean age of 25.3 years ± 4.458) were tasked with classifying facial images, and only those photographs for which consensus was reached by more than 70% of the students were selected for further analysis (Seo et al., 2020) (see Supplementary). To ensure the random presentation of the photographs, a Python program was developed to display them alongside a background screen intermittently.

#### 2.2.2 Delay discounting

Monetary Choice Questionnaire (MCQ) has been demonstrated to be a valid and reliable instrument for assessing delay discounting (Kirby et al., 1999; Strickland et al., 2023). Participants were presented with nine binary choice items, requiring them to select between smaller, immediately available monetary amounts (e.g., $11 today) and larger, delayed rewards (e.g., $30 in 7 days). To quantify the preference for immediate versus delayed rewards, a hyperbolic discount rate (*k*) was calculated using the formula V = A/(1 + *k*D), where V signifies the value of the immediate reward, A represents the delayed reward value, D indicates the duration of the delay in days, and *k* serves as the free parameter that reflects the degree of discounting. The range of values for *k* in the MCQ-9 questionnaire is from 0.00016 to 0.25, with higher values indicating a stronger preference for immediate rewards over delayed rewards. The estimation of the patient’s discounting rate *k* involves calculating the geometric midpoint between the highest *k* value associated with the item chosen for immediate reward and the lowest *k* value associated with the item chosen for delayed reward. This calculation helped determine the participant’s point of indifference between immediate and delayed rewards.

For instance, in question 1, participants were presented with the option of receiving “$54 today” or “$55 in 117 days”. The discount rate (*k*) associated with this item is calculated to be 0.00016, indicating that a participant with a discount rate of 0.00016 would be indifferent between these two rewards. Consequently, if a participant opted for the immediate reward in this scenario, it can be inferred that their discount rate exceeds 0.00016. Similarly, in question 2, participants were given a choice between “$47 today” and “$50 in 160 days”, with a corresponding *k* value of 0.0004. Hence, if the same participant were to select the delayed reward in this particular trial, it would suggest that their discount rate is below 0.0004. Considering both trials, it can be inferred that their discount rate falls within the range of 0.00016 to 0.0004. Therefore, the participant’s *k* value was estimated to be 0.00025 by calculating the geometric mean of this interval.

### 2.3 Procedures

#### 2.3.1 Pre-operative interview

Prior to the pre-operative interview, each participant received a comprehensive explanation regarding the purpose and nature of the current investigation and signed the informed consent. All participants underwent interviews at least 30 min before GI, during which baseline data were gathered. This included demographic information such as age, gender, height, weight, socio-economic status (indicated by highest educational degree and years of education), past medical history, comorbidities, smoking and alcohol consumption history, and the valid sleep time before the surgery day. Given that depression (Gao et al., 2019), anxiety (Tseng et al., 2017), insomnia (de Almondes et al., 2020), and alcohol addiction (Castellano et al., 2015) can potentially impact FER and delay discounting, additional evaluations were conducted on patients who presented the following complaints without seeking medical attention: for patients who reported feeling “not in a good mood recently,” the Patient Health Questionnaire-9 (PHQ-9) was administered, and a score of ≥5 was recorded to indicate a state of depression (Collins et al., 2022). For patients who reported feeling “intense or anxious recently,” the Beck Anxiety Inventory (BAI) was utilized, and a score of ≥15 was recorded to indicate a state of anxiety (Suzen Ozbayrak et al., 2021). For patients who reported experiencing difficulties with sleep recently, the Pittsburgh Sleep Quality Index (PSQI) was employed, and a score of ≥6 was recorded to indicate recent insomnia (Zhou et al., 2022). For patients who reported recent alcohol consumption, the Alcohol Use Disorder Identification Test (AUDIT) was used, with a score of ≥8 as indicative of alcohol addiction was recorded (Neufeld et al., 2021). The FER and delay discounting were assessed as previously described. All the psychology and behavioral assessments were conducted by trained evaluators.

#### 2.3.2 Perioperative management

The administration of general anesthesia was carried out by two anesthesiologists (Z. S and Q. L). Perioperative management, encompassing anesthetic practice and perioperative care, adhered to the institutional protocols based on current guidelines and expert consensus (Lee and Lee, 2014).

Before entering the operating room, intravenous catheterization was performed, and Ringers lactate solution was infused in the preparation room. Upon transfer to the operating room, non-invasive blood pressure measurements were taken every 3 min, along with electrocardiograms, heart rate (HR), pulse oxygen saturation (SpO_2_), and bispectral index (BIS) monitoring throughout the entire procedure. Oxygen at a rate of 5 L/min was delivered via nasal catheter. For anesthesia induction, patients were administered fentanyl at a dosage of 0.001 mg/kg and propofol at a dosage of 1.5–2.5 mg/kg, with an infusion rate of 800 mL/h. For anesthesia maintenance, propofol was continuously infused at a dosage of 4–8 mg/kg/h, with BIS maintained between 40 and 65 (Johansen and Sebel, 2000).

Treatment and dosage adjustment were administered in accordance with clinical requirements. Extra propofol was administered where the patient exhibited gag reflex, coughing, body movement, or a BIS ≥65. If the administration of propofol failed to alleviate the aforementioned reflexes, an additional dosage of fentanyl was administered, and the patient was subsequently excluded from the data analysis. Additional treatments included assisted ventilation through a facial mask when the patient’s SpO_2_ (oxygen saturation) fell below 90%, administration of atropine at 0.5 mg when the patient’s heart rate dropped below 45 bpm, and the administration of either ephedrine at 5–10 mg or dopamine at 1–2 mg when the patient’s systolic non-invasive blood pressure fell below 90 mmHg.

The procedures were performed once the patients’ eyelash reflex disappeared. All endoscopic procedures were carried out by experienced endoscopists with a minimum of 5 years of experience in performing colonoscopies as the guidelines instructed (Qiu et al., 2022). The propofol infusion was discontinued once the colonoscopy had reached the ileocecal area and was ready for retraction. After the examination, patients were tapped on the shoulder. Once a modified Aldrete score of 8 was reached, patients were transferred to the post-anesthesia care unit for additional recovery.

#### 2.3.3 Postoperative assessment

After patients fully recover from anesthesia and before discharge, approximately half an hour after GI endoscopy, they were also assessed for FER and delay discounting, as well as for their sense of well-being and dream content.

### 2.4 Sample-size estimation

To the best of available knowledge, there is a lack of research on the impairment of FER or delay discounting after GI with propofol anesthesia. A pilot study, consisting of 35 patients, revealed that the accuracy of FER for anger was 48.6% pre-GI, compared to 32.4% post-GI. To ensure statistical significance with a two-sided *α* value of 0.05 and 85% power, accounting for a 5% drop-out rate, 104 patients were planned to be enrolled in this investigation.

### 2.5 Data analysis

The normal distribution of variables was assessed using the Kolmogorov–Smirnov test. Continuous data with a normal distribution were presented as mean ± standard deviation (SD) and analyzed using the *t*-test or paired *t*-test, as appropriate. Data that did not follow a normal distribution were presented as median ± interquartile range and were analyzed using the Wilcoxon signed-rank test. Categorical variables were presented as number (%) and compared using the chi-square test or McNemar’s test. A multiple linear regression analysis was conducted to investigate the factors influencing impaired FER of anger. All statistical analyses were performed using SPSS version 25.0. Statistical significance was defined as a *p*-value less than 0.05.

## 3 Results

### 3.1 Demographic characteristics and perioperative conditions

**Figure 1:**
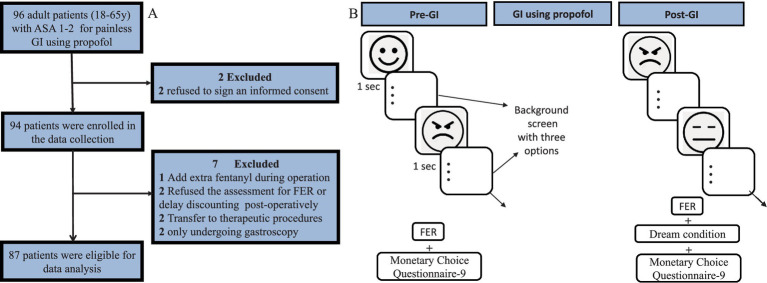
Study flow chart **(A)** and data collection procedure **(B)**. This study employed a tablet-based Facial Expression Recognition task. Nine grayscale photographs were presented randomly on the tablet screen. Each face was displayed for a duration of one second, followed by a background screen displaying visual white noise. During this interval, patients were instructed to select the most appropriate emotion label from three options (Happiness, Anger, or Neutral) using a provided form. There was no time limit for responding, but patients were encouraged to provide their initial impression as quickly as possible. To ensure the random presentation of the photographs, a Python program was developed to display them alongside a background screen intermittently. A correct answer was assigned a score of 1, while an incorrect answer received a score of 0. The scoring system ranged from 0 to 3 for each expression. ASA, American Society of Anesthesiologists; GI, gastrointestinal endoscopy; FER, facial emotional recognition.

A total of 96 patients were initially screened for inclusion in the study. Ultimately, 87 patients met the eligibility criteria and were included in the data analysis (Figure 1). The patients’ demographic characteristics, perioperative conditions, and management are presented in Table 1.

**Table 1:** The demographic characteristics and the perioperative management variables.

Variables	Value
Preoperative characteristics	
Age, years	45.3 ± 10.12
Gender, Female	47 (54.0%)
Height, cm	166.9 ± 7.96
Weight, kg	64.93 ± 12.28
BMI	23.16 ± 3.36
Pre-operative valid sleep time, h	6.0 ± 3.5
Highest level of education	
Doctor’s degree	9 (11.25%)
Master’s degree	19 (23.75%)
Bachelor’s degree	30 (37.5%)
Junior college	13 (16.25%)
Senior middle school	4 (5%)
Junior middle school	5 (6.25%)
Medical and personal history	
Hypertension	9 (10.34%)
Coronary heart disease	2 (2.30%)
Diabetes mellitus	5 (5.75%)
Hyperlipidemia/hypercholesterolemia	9 (10.34%)
Cerebrovascular disease	1 (1.15%)
Recent insomnia	8 (9.20%)
State or trait depression	6 (6.90%)
State or trait anxiety	5 (5.75%)
Recent smoking	10 (11.49%)
Alcoholism	5 (5.75%)
Intraoperative management	
Cases undergoing GI in different time period, 8:00–12:00 (vs 13:00–17:00)	52 (59.8%)
Duration of propofol infusion, min	18.28 ± 4.70
Total propofol infusion, mg	215.0 ± 60.0
propofol consumption per min per kilogram body weight, mg kg^−1^ min^−1^	0.20 ± 0.05
Cases administering extra propofol	62 (71.3%)
Intraoperative adverse reactions (cases occurred)	
SBP < 90 mmHg	8 (9.2%)
HR < 45 bpm	0
BIS<40	5 (5.7%)
SpO_2_ < 90%	0

GI, gastrointestinal endoscopy; SBP, systolic blood pressure; HR, heart rate; BIS, Bispectral index; SpO_2_: Saturation of pulse oxygen; Data are presented as mean ± SD, median ± interquartile range, or number (percentage), as appropriate.

### 3.2 Changes in facial emotion recognition (FER) scores after propofol anesthesia

**Figure 2:**
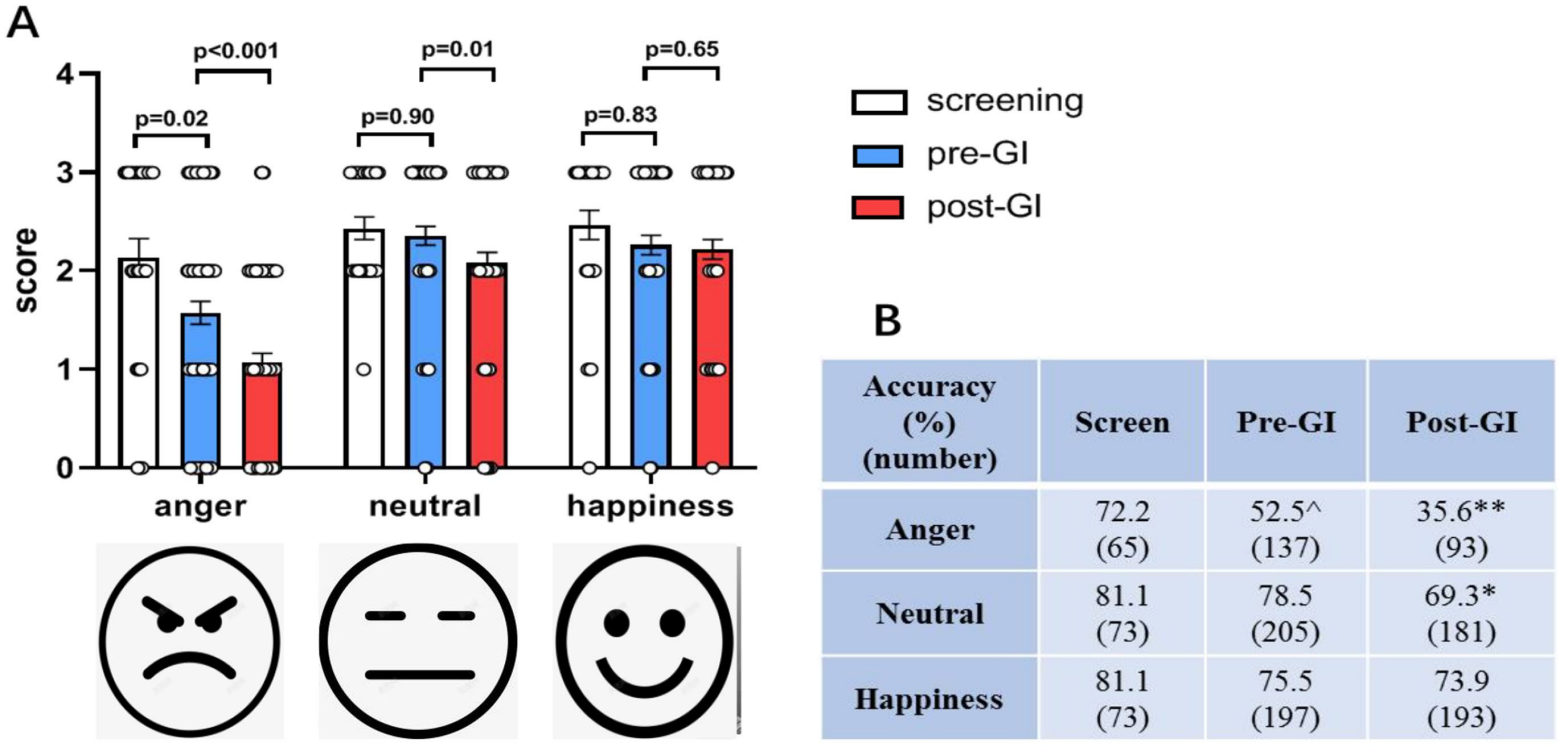
The mean score and accuracy of FER of anger and neutral expressions decreased post-GI using propofol. **(A)** Compared to pre-GI, the score of FER of angry and neutral expressions dramatically decreased post-GI (*n* = 87). There was no significant difference between pre and post-GI on the scores of FER of happiness. The patients’ scores of FER of anger pre-GI were significantly different from those of college students for screening. **(B)** The statistical results of the accuracies were consistent with the scores (*n* = 30). GI: gastrointestinal endoscopy. Data are presented as mean ± SEM. ^*p* < 0.01 vs. the screen group; *p < 0.01 and **p < 0.001 vs. the pre-GI group.

According to the 1SD criterion, 11 of 87 patients (12.6%) met the criterion of FER deficit. In order to examine the potential impact of propofol anesthesia on FER, assessments of positive (happiness), negative (anger), and neutral expressions were conducted pre- and post-GI. The analysis of FER includes three aspects: the mean score and accuracy, the FER variation between pre- and post-GI, considering both correct and incorrect recognition, and the trend of incorrect FER variation. Compared to pre-GI, the FER score of anger and neutral significantly decreased post-GI. However, there was no significant difference in the FER scores of happiness between pre and post-GI (Figure 2A). Additionally, it is worth noting that the patients’ scores and accuracy of FER of anger pre-GI were significantly different from those obtained from college students during screening, while the FER scores and accuracy of FER of neutral and happiness pre-GI showed no differences with those of screening (Figure 2A). The statistical results of the accuracies were consistent with the scores (Figure 2B).

### 3.3 The percentage of patients with correct and incorrect FER before and after gastrointestinal endoscopy

**Figure 3:**
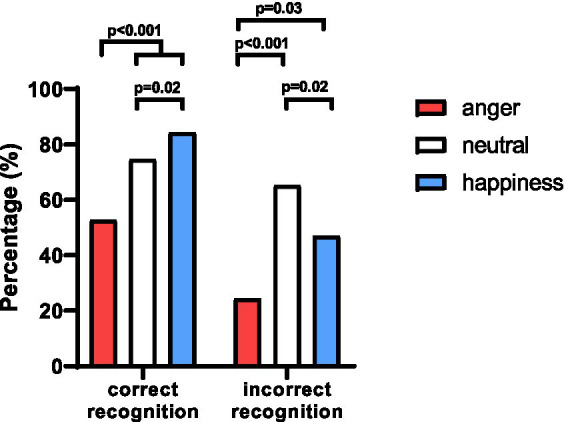
Variation of FER pre- and post-GI, considering both the correct and incorrect answers. No matter correctly (52.6%) or incorrectly (24.3%) recognized facial emotion as anger, the percentage to maintain the same choices of anger post-GI was notably lower than the percentages of neutral and happiness. Additionally, the percentage of participants who maintained correct identification of happiness was higher than that of neutral (*p* = 0.02), while the percentage of incorrect choices for happiness was lower than that of neutral (*p* = 0.02).

Given that the accuracy of emotion recognition has been found to be influenced by factors such as education (Trauffer and Russell, 2013), living environment (da Silva Ferreira et al., 2014), and current mood (Beukeboom and Semin, 2005; Forgas and East, 2008),and as evidenced by prior research, the changes between FER pre- and post-GI were also compared by considering both correct and incorrect recognition. Among patients who correctly recognized facial emotions pre-GI, half (52.6%) maintained their choices of anger post-GI, which was notably lower than that of neutral (74.6%, *p* < 0.001) and happiness (84.3%, *p* < 0.001), which met two thirds or more patients. Among patients who initially misidentified facial emotions as anger pre-GI, less than one quarter (24.3%) continued to choose anger post-GI, which was significantly lower than that of neutral (65.2%, *p* < 0.001) or happiness (46.9%, *p* = 0.03). Additionally, the percentage of incorrect choices for happiness was lower than that of neutral (*p* = 0.02) (Figure 3).

### 3.4 Identification bias for FER after propofol anesthesia during gastrointestinal endoscopy

Then, we determined which expression would be more prone to be incorrectly identified for FER after propofol anesthesia. The number of patients with incorrect identification of the 3 facial expressions pre- and post-GI endoscopy was compared using Chi-square test. The results revealed that compared to pre-GI, patients were more susceptible to incorrectly identify anger (*p* = 0.02) and neutral (*p* = 0.01) expressions as happiness post-GI (Table 2). However, there were no differences for FER of anger being incorrectly identified as neutral (*p* = 0.07) or for neutral being incorrectly identified as anger (*p* = 0.34) when comparing pre-GI with post-GI. Moreover, no significant differences were observed in the incorrect identification of happiness as the other two expressions, comparing pre-GI with post-GI (Table 2).

**Table 2:** Difference of incorrect identification expression pre- and post-GI (number of patients).

Expression	Incorrect identification expression	Pre-GI	Post-GI	*p*-value
Anger	Happiness	17	32	**0.02**
	Neutral	107	128	0.07
Neutral	Happiness	32	53	**0.01**
	Anger	34	27	0.34
Happiness	Neutral	51	57	0.52
	Anger	13	11	0.68

Bold values indicate predictors with statistical significance (*p* < 0.05).

### 3.5 Factors associated with impaired anger recognition after GI endoscopy

The results above suggested that propofol anesthesia impaired more on FER of anger. Therefore, an investigation was conducted to identify the factors contributing to the discrepancy in anger FER scores between pre- and post-GI. Univariable linear regression analysis indicated associations (*p* < 0.2) between various factors, including height, diabetes mellitus, state or trait anxiety, recent insomnia, different periods of GI, and the times of administering extra propofol, with the differences in FER scores between pre- and post-GI (Supplementary 1). A subsequent multiple linear regression analysis was performed, showing that undergoing the procedure in the morning (*p* = 0.006) and the absence of insomnia (*p* = 0.01) significantly lowered the score and accuracy of FER of anger post-GI (Table 3). It is noteworthy to mention that three other factors, namely state or trait anxiety (*p* = 0.08), height (*p* = 0.08), and the times of administering extra propofol during GI (*p* = 0.054) exhibited marginal significance (Table 3). This suggests that with an increased sample size, these variables may be associated with the score of FER of anger post-GI.

**Table 4:** Postoperative dream conditions and sense of well-being.

Postoperative interview	Values (*n*[%])
Having dream	25 (28.7%)
Dream content being able to recall	15 (17.2%)
Dream content, good (vs bad or general)	13 (14.9%)
Sense of well-being	45 (51.7%)

**Table 3:** Multiple linear regression analysis of the differences in FER scores between pre- and post-GI and related factors.

Variables	Unstandardized coefficients		*t*	*p*-value	VIF
	B	Std. error			
Height	0.024	0.014	1.775	0.080	1.038
Diabetes mellitus	−0.434	0.464	−0.936	0.352	1.036
State or trait anxiety	0.851	0.480	1.774	0.080	1.110
Recent Insomnia	−1.015	0.386	−2.629	**0.010**	1.106
Undergoing GI in different time period	−0.626	0.221	−2.826	**0.006**	1.031
Number of times administering extra propofol	0.252	0.129	1.956	0.054	1.065

Bold values indicate predictors with statistical significance (*p* < 0.05).

### 3.6 Delay discounting behavior after propofol anesthesia during GI endoscopy

**Figure 4:**
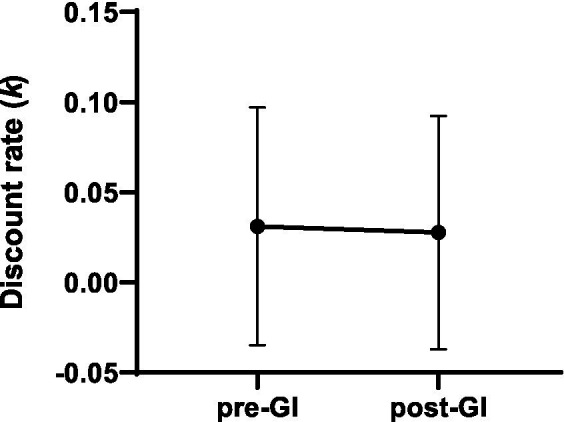
Propofol anesthesia did not impair delay discounting behavior, tested by Monetary Choice Questionnaire-9. *K*-value showed no difference between pre- and post-GI. Data are presented as mean ± SEM.

To determine the impact of propofol anesthesia on delay discounting behavior, patients’ delay discounting was further investigated using MCQ-9 pre and post-GI. There was no difference in *k* value between pre- and post-GI (*p* = 0.07), suggesting that propofol anesthesia did not impair the patient’s delay discounting behavior in this study (Figure 4).

### 3.7 Investigation of dream conditions and sense of well-being during GI with propofol

As propofol has been associated with the experience of pleasant dreams and a sense of well-being after use (Zhao et al., 2023; Qiu et al., 2022; Brechmann et al., 2018), dream conditions were also investigated during the post-GI interview. Out of the total population, 25 patients (28.7%) reported having dreams, among which 60% (15, 17.2% of the total population) were able to recall the content of their dreams. Among those who could recall their dream content, 86.7% (13, 14.9% of the total population) experienced pleasant dreams. The remaining two patients described having ordinary dreams related to daily living. No instances of bad dreams were reported. Approximately half (45, 51.7%) of the total population reported a sense of well-being, mostly characterized by smiling, grinning, and even laughing right after wake up (Table 4). Furthermore, a comparison of pre- and intra-operative factors between dreamers and non-dreamers was conducted. The results showed that younger patients exhibited a greater propensity to dream during propofol anesthesia (Table 5). Additionally, it is worth noting that the variable “cases in different periods” demonstrated marginal significance (*p* = 0.06), suggesting that with an increased sample size, the time period may be associated with dreaming after GI using propofol (Table 5).

**Table 5:** Pre- and intra-operative factors between dreamers and non-dreamers.

Variables	Dreamers (*n* = 25)	Non-dreamers (*n* = 62)	*p*-value
*Pre-operative baselines*			
Age, years	41.92 ± 11.36	46.66 ± 9.33	**0.047**
Gender, female	12 (48%)	35 (56.5%)	0.47
Body Weight Index (BMI)	22.36 ± 3.36	23.51 ± 3.31	0.15
Pre-operative valid sleep time, h	5.75 ± 2.43	5.37 ± 2.00	0.53
*Intraoperative conditions*			
Propofol consumption mg^−1^ kg^−1^ min^−1^	0.21 ± 0.05	0.20 ± 0.05	0.28
Cases undergoing GI in different period, 8:00–12:00 (vs 13:00–17:00)	11 (44%)	41 (66.1%)	0.06
Cases administering extra propofol	21 (84%)	41 (66.1%)	0.10

Data are presented as mean ± SD and number (percentage), as appropriate.

Bold values indicate predictors with statistical significance (*p* < 0.05).

## 4 Discussion

This study is to investigate the impact of propofol anesthesia on social cognitive functions, such as emotion recognition and risk decision-making. The study indicates that propofol anesthesia administered during GI has a more detrimental effect on the FER of anger expressions than that of neutral and happiness expressions. Moreover, a positive bias was observed for FER of post-GI anger and neutral expressions. The impairment in anger recognition post-GI was found to be associated with GI conducted in the morning and the absence of insomnia. Propofol anesthesia did not impact FER of happiness and delay discounting behavior. In this study, the incidence of FER deficit was 12.6%. In contrast, two previous studies investigating emotion recognition after cardiac surgery reported an FER deficit incidence of 1/5–1/3 (Zhang et al., 2020, 2022). The discrepancy in these findings may be attributed to several factors that can impair neurocognitive function, such as prolonged surgical duration, increased surgical trauma, and variations in the types and dosages of anesthetics used during anesthesia induction and maintenance of cardiac surgery.

While previous studies, such as those by Zhang et al. (2020, 2022), have laid important groundwork, certain methodological limitations remain. In the 2020 study, white dots were displayed on the black background to form an outline of a human figure in motion (e.g., walking, jumping). With randomly moving dots, observers with normal social perception abilities were able to differentiate the movement from randomly-moving-dot noises. Their 2022 follow-up study expanded this to include three emotional expressions (anger, happy and sad) within the point-light paradigm. However, a key limitation was that the analysis assessed the recognition accuracy collectively without differentiating between specific emotions, which may obscure expression-specific effects. To enable a more nuanced investigation, the present study analyzed the FER results of different expressions through three perspectives, including overall accuracy, variation in FER considering both correct and incorrect recognition, and the trend of variation in incorrect FER.

The findings in this study indicate that, in comparison to neutral and happiness, the FER of anger is more susceptible to impairment by propofol anesthesia during GI. These results align with previous studies demonstrating that the vulnerability of FER for negative emotions is easily affected by biological and pathological factors, while happiness appears to be less affected (Ciccarelli et al., 2022; da Silva Ferreira et al., 2014). For example, one meta-analysis revealed widespread impairments in facial and vocal recognition, with notable deficits in recognizing negative emotions among adults and children/adolescents with psychopathy (Dawel et al., 2012). Additional studies demonstrated that individuals with personality disorders and anxiety disorders performed poorly in visually recognizing negative emotions such as anger, fear, and sadness (Ciccarelli et al., 2022; Chuang et al., 2021). Furthermore, research indicated that maltreated children exhibited reduced accuracy and response bias in recognizing faces displaying negative emotions, particularly anger (da Silva Ferreira et al., 2014). On the other hand, a study has shown that the easiest identified emotion is happiness for both dementia patients and healthy controls (Guaita et al., 2009). Collectively, these studies suggest that negative emotions, particularly anger, are more susceptible to impairment. This study found a positive identification bias in FER of anger and neutral expressions post-GI. It is well demonstrated that propofol administration may induce euphoria, elation, and a sense of well-being. Further research is necessary to determine if there is a shared mechanism between propofol anesthesia and FER impairment.

The study further examined the factors that impaired FER of anger after propofol anesthesia. The findings revealed that undergoing GI in the morning and the absence of insomnia significantly reduced the accuracy and score of anger recognition post-GI. To the best of available knowledge, no existing study has examined the relationship between various time periods and FER. However, studies have been conducted on different chronotypes and FER, demonstrating a negative bias in emotion processing among individuals with a late chronotype (commonly referred to as “Owls” who prefer to sleep and wake up late), comparing to morning types (referred to as “Larks” with early rise and bedtimes). Further research is needed to elucidate the underlying mechanisms. This study found that recent insomnia can positively impact the accuracy of FER of anger, which might indicate a potential therapeutic effect of propofol (de Almondes et al., 2020). However, the results did not reveal any significant interaction between the doses of propofol administered or the difference in BIS and FER post-GI. To gain a more comprehensive understanding of the relationship between the administration of additional propofol and FER, further investigations with a larger sample size are warranted. The study conducted by Zhang et al. also analyzed factors influencing social cognition impairment and concluded that age, gender, or education level do not significantly affect social cognition (Zhang et al., 2022). The present findings corroborated these findings.

Additionally, it was demonstrated that propofol anesthesia did not impair delay discounting. This is consistent with previous studies indicating that adolescents with substance (tobacco, marijuana, or alcohol) abuse exhibited vulnerability towards the FER of negative emotion but not towards decisions that involve risk (Ernst et al., 2010). Given that propofol has been associated with addictive potential, additional investigation is warranted to elucidate the underlying mechanisms responsible for the differential effects on FER and delay discounting.

There are several limitations in this study. Firstly, an extended follow-up duration after anesthesia and surgery should have been implemented, since it is essential to assess the long-term effects of social cognitive impairment on quality of life. Furthermore, only two specific emotions (anger and happiness) were examined, more researchers are encouraged to explore additional emotions in their investigations. Thirdly, the potential presence of learning effects in the FER task and MCQ-9 assessment post-GI cannot be ruled out despite efforts to minimize this effect through the random display of photographs. Fourthly, as only Chinese patients were enrolled in this study, the results may have limited generalizability due to the demographic composition. Therefore, further research is needed to validate these findings.

## 5 Conclusion

Overall, this study demonstrated that propofol anesthesia was associated with a decrease in anger recognition while leaving the recognition of happiness and delay discounting unaffected, as observed shortly after gastrointestinal endoscopy. Additionally, the findings indicate a positive bias in FER after propofol anesthesia. These results provide a novel perspective on postoperative social cognitive function and highlight the need for increased focus on social cognitive dysfunction after surgery and anesthesia. Further research is needed on the mechanisms underlying prolonged impairment after propofol anesthesia.

## Data Availability

The raw data supporting the conclusions of this article will be made available by the authors, without undue reservation.
