# Supplementary material for: Selective impairment and a positive recognition bias of the facial emotion recognition after propofol anesthesia during gastrointestinal endoscopy
Source: Front Psychol. 2026 Jan 13;16:1691042. doi: 10.3389/fpsyg.2025.1691042 (PMC12834756; doi:10.3389/fpsyg.2025.1691042)
Supplement: Supplementary file 1 [file Table_1.docx]

**Supplementary 1. Univariable linear regression analysis of differences in FER scores between pre- and post-GI and related factors.**

| Variables | Unstandardized Coefficients | | t | p-value |
| --- | --- | --- | --- | --- |
|  | B | Std.Error |  |  |
| Age | -0.008 | 0.012 | -0.695 | 0.489 |
| Gender | 0.057 | 0.235 | 0.242 | 0.809 |
| Height | 0.030 | 0.015 | 2.040 | **0.044** |
| Weight | 0.010 | 0.010 | 0.997 | 0.321 |
| BMI | 0.003 | 0.036 | 0.092 | 0.927 |
| Pre-operative valid sleep time | -0.032 | 0.071 | -0.456 | 0.650 |
| Years of education | 0.011 | 0.038 | 0.291 | 0.772 |
| Hypertension | -0.440 | 0.382 | -1.152 | 0.253 |
| Coronary heart disease | -0.006 | 0.783 | -0.008 | 0.994 |
| Diabetes Mellitus | -0.749 | 0.497 | -1.505 | **0.136** |
| Hyperlipidemia/ Hypercholesterolemia | 0.056 | 0.385 | 0.144 | 0.886 |
| Cerebrovascular disease | 0.500 | 1.099 | 0.455 | 0.650 |
| State or trait anxiety | 0.737 | 0.498 | 1.480 | **0.143** |
| Recent Insomnia | -0.832 | 0.396 | -2.103 | **0.038** |
| State or trait Depression | -0.185 | 0.462 | -0.400 | 0.690 |
| Recent smoking | -0.006 | 0.368 | -0.018 | 0.986 |
| Alcoholism | -0.537 | 0.501 | -1.072 | 0.287 |
| Minimal value of SBP | 0.010 | 0.009 | 1.104 | 0.273 |
| Difference between the highest  and lowest SBP | 0.004 | 0.007 | 0.643 | 0.522 |
| Minimal value of HR | 0.006 | 0.014 | 0.472 | 0.638 |
| Difference between the highest  and lowest HR | -0.016 | 0.013 | -1.196 | 0.235 |
| Minimal value of BIS | 0.002 | 0.014 | 0.162 | 0.872 |
| Total propofol infusion | 0.008 | 0.022 | 0.381 | 0.704 |
| Duration of propofol infusion | -0.004 | 0.026 | -0.155 | 0.877 |
| propofol consumption per min  per kilogram body weight | -0.470 | 2.423 | -0.194 | 0.847 |
| Times of administering  extra propofol | 0.188 | 0.133 | 1.414 | **0.161** |
| Undergoing GI in different  time period | -0.512 | 0.233 | -2.198 | **0.031** |
| Having dreams | 0.020 | 0.259 | 0.077 | 0.939 |
| Dream content able to recall | 0.194 | 0.310 | 0.628 | 0.532 |
| Dream content, good vs others | 0.400 | 0.326 | 1.227 | 0.223 |
| Sense of well-being | 0.057 | 0.235 | 0.244 | 0.808 |

BMI, Body Mass Index; SBP, systolic blood pressure; HR, heart rate; BIS, Bispectral index; SpO_2_, Saturation of pulse oxygen
